# In Situ Growth of MoS_2_ Onto Co‐Based MOF Derivatives Toward High‐Efficiency Quantum Dot‐Sensitized Solar Cells

**DOI:** 10.1002/advs.202406476

**Published:** 2024-09-16

**Authors:** Tianming Wang, Lejuan Cai, Caijuan Xia, Han Song, Lianbi Li, Gongxun Bai, Nianqing Fu, Lede Xian, Rong Yang, Haoran Mu, Guangyu Zhang, Shenghuang Lin

**Affiliations:** ^1^ School of Science Xi'an Polytechnic University Xi'an Shanxi 710048 China; ^2^ Songshan Lake Materials Laboratory Dongguan Guangdong 523808 China; ^3^ College of Chemistry and Chemical Engineering Xinjiang Normal University Xinjiang Uygur Autonomous Regions Urumqi 830054 China; ^4^ Key Laboratory of Rare Earth Optoelectronic Materials and Devices of Zhejiang Province China Jiliang University Hangzhou 310018 China; ^5^ School of Materials Science and Engineering South China University of Technology Guangzhou 510641 China; ^6^ Changsha Semiconductor Technology and Application Innovation Research Institute College of Semiconductors (College of Integrated Circuits) Hunan University Changsha 410082 China

**Keywords:** Co,N─C@MoS_2_ composite, counter electrode, high efficiency, quantum dot sensitized solar cells

## Abstract

Quantum dot sensitized solar cells (QDSCs) represent a promising third‐generation photovoltaic technology, boasting a high theoretical efficiency of 44% and cost efficiency. However, their practical efficiency is constrained by reduced photovoltage (*V*_oc_) and fill factor (FF). One primary reason is the inefficient charge transfer and elevated recombination rates at the counter electrode (CE). In this work, a novel CE composed of a titanium mesh loaded with Co,N─C@MoS_2_ is introduced for the assembly of QDSCs. The incorporation of nanosized MoS_2_ enhances the density of catalytic sites, while the Co,N─C component ensures high conductivity and provides a substantial active surface area. Additionally, the titanium mesh's 3D structure serves as an effective electrical conduit, facilitating rapid electron transfer from the external circuit to the composite. These improvements in catalytic activity, charge transfer rate, and stability of the CE significantly enhance the photovoltaic performance of QDSCs. The optimized cells achieve a groundbreaking power conversion efficiency (PCE) of 16.39%, accompanied by a short‐circuit current density (*J*_sc_) of 27.26 mA cm^−2^, *V*_oc_ of 0.818 V, and FF of 0.735. These results not only offer a new strategy for designing electrodes with high catalytic activity but also underscore the promising application of the Co,N─C@MoS_2_ composite in enhancing QDSC technology.

## Graphical abstract

A novel titanium mesh loaded with Co,N─C@MoS_2_ composite counter electrode (CE) is developed for high‐efficiency quantum dot‐sensitized solar cells (QDSCs). In situ chemical vapor deposition technology is used to prepare the composite. The obtained CEs illustrate excellent catalytic activity, conductivity, hydrophilicity, charge transfer rate, and stability. The ZCISSe QDSCs based on Co,N─C@MoS_2_/Ti CEs achieve a high PCE of 16.39%.

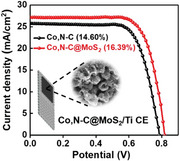

## 1 Introduction

Quantum dot‐sensitized solar cells (QDSCs) are a type of high potential, low‐cost third‐generation solar cells that use semiconductor nanocrystals (quantum dots, QDs) as light capture materials.^[^ ^1^ ^]^ Due to the high absorption coefficient, adjustable absorption range, simple preparation process, low cost, and multi‐exciton effect of QDs,^[^ ^2^ ^]^ QDSCs have significant advantages over traditional solar cells.^[^ ^3^ ^]^ Due to the development of QD sensitizers with wide spectral absorption, high conduction band energy potential, efficient adsorption methods, and the application of passivation surface engineering to suppress electron recombination technology,^[^ ^4^, ^5^, ^6^ ^]^ the reported highest photoelectric conversion efficiency of QDSCs has reached 16%,^[^ ^7^ ^]^ which breaking the 15% efficiency threshold required for commercial applications of solar cells. However, the highest reported efficiency is still far less than the corresponding theoretical value (44%), as well as there are still many key scientific problems to be solved. The short‐circuit current density (*J*_sc_ ≈ 26 mA cm^−2^) is already comparable to other light‐absorbing materials such as dye sensitizers and perovskite materials.^[^ ^8^, ^9^, ^10^ ^]^ Unfortunately, the relatively low photovoltage (*V*_oc_ < 0.80 V) and filling factor (FF < 0.75) limit the further improvement of the efficiency of QDSCs.^[^ ^11^, ^12^ ^]^

The unsatisfactory photovoltage value is mainly attributed to the relatively high redox potential of polysulfide electrolytes (S_n_^2−^/S^2−^), which requires a high overpotential for the reduction of electrons obtained from the electrolyte in QDs.^[^ ^13^, ^14^ ^]^ Research reports have proposed that modified polysulfide electrolyte can suppress the charge recombination process of QDSCs effectively, thereby increasing the photovoltage of device.^[^ ^15^ ^]^ The lower FF value is mainly related to the lower catalytic activity of the counter electrode (CE) currently used, leading to the lower catalytic reduction reaction of the electrolyte and the energy loss of carriers during the transfer process. Relatively speaking, it is possible to improve the performance of QDSCs by simply regulating the catalytic activity of CEs to enhance FF. As an important component of QDSCs, the main role of CEs is to collect external circuit electrons and transfer them to electrolyte through catalytic reduction of the oxidized electrolyte. The ideal electrode material should meet the following characteristics: 1) simple preparation process and low cost; 2) good conductivity and high catalytic activity; 3) excellent chemical stability. Therefore, it is the key to select highly comprehensive performance CE materials to enhance the efficiency of QDSC.

The CEs of QDSCs mainly consist of two parts: catalytic materials and conductive substrates that load the catalytic material. At present, the commonly used CE substrates include Cu_2_S/brass, FTO conductive glass, and titanium mesh.^[^ ^16^, ^17^, ^18^, ^19^ ^]^ Among them, Cu_2_S/brass is difficult to package treatment and easily corroded by polysulfide electrolyte, while FTO substrate has poor conductivity. Moreover, the catalytic material is not firmly adhered to the FTO glass, resulting in detaching from the substrate. These factors to some extent limit the development of QDSCs. In 2017, Zhong's group first proposed Ti mesh‐supported mesoporous carbon (MC/Ti) CE.^[^ ^20^ ^]^ Ti mesh substrate can anchor MC catalytic material in the grid, which offers robust carbon film with millimeter thickness to ensure high catalytic capacity effectively. In addition, the 3D electrical tunnel of Ti mesh could improve the electron transport ability and ensure high catalytic performance of the CEs. Therefore, Ti mesh substrate is currently considered to be the best conductive substrate for QDSCs CEs.

Carbon materials have been explored as catalytic materials of CEs for QDSCs due to their excellent electrocatalytic activity, conductivity, high stability, and low cost.^[^ ^21^ ^]^ Currently, a series of carbon materials, such as MC, nitrogen‐doped mesoporous carbon (NMC), Co,N co‐doped mesoporous carbon (Co,N─C), graphene hydrogel, and carbon nanotubes, have been reported and confirmed to have a good catalytic effect on polysulfide electrolyte in QDSCs.^[^ ^21^, ^22^, ^23^ ^]^ Moreover, QDSCs achieved over 16% power conversion efficiency (PCE) with NMC/Ti CEs under high QDs loading photoanodes.^[^ ^7^ ^]^ In a recent work by Zhong and co‐workers, quasi‐2D sheet‐like Co,N─C materials were prepared through the pyrolysis treatment of the CoAl‐LDH@ZIF‐67 composite. The materials are beneficial to increase the specific surface area and improve the permeability of the polysulfide electrolyte and therefore facilitate the exposure of the catalytic active sites and enhance the catalytic activity of the CEs. As a result, an average PCE of 13.55% was achieved for ZCISe QDSCs based on Co,N─C/Ti CE.^[^ ^24^ ^]^ Similarly, Co,N─C/Ti CEs were also used to construct the CdSe QDSCs, and a high cell efficiency of 6.85% was obtained.^[^ ^25^ ^]^ However, the above‐mentioned carbon‐based catalytic materials cannot simultaneously meet the requirements of highly electrocatalytic activity and chemical stability. Moreover, charge transfer to the electrode and charge recombination are also key factors in improving PCE of QDSCs. In other words, preparing electrodes with high catalytic activity and high charge transfer performance will help reduce charge recombination and improve device efficiency.

MoS_2_ crystal, as a classic 2D material in transition metal sulfides (TMDs), has a typical layered structure and many catalytic active sites.^[^ ^26^, ^27^ ^]^ The layers are connected by weak van der Waals forces and can be easily separated into single or multiple layers. In addition, it possesses the characteristics of abundant reserves, low cost, adjustable bandgap, and band position, and has been widely studied in energy storage and conversion devices (lithium‐ion batteries, dye‐sensitized solar cells, capacitors, etc.).^[^ ^28^, ^29^, ^30^ ^]^ However, there are few literatures on the research of MoS_2_ materials in QDSCs. It is found that MoS_2_ material has a 2D layered structure similar to graphene, and its catalytic activity depends on the size, number of layers, and number of edge active sites.^[^ ^31^ ^]^ The catalytic performance of MoS_2_ could be improved by increasing the number of edge active sites.^[^ ^32^, ^33^ ^]^ The previous paper reported that the ultrathin 2D nanostructure of MoS_2_ illustrated a high specific surface area and good electronic transport path.^[^ ^34^ ^]^ The exposed sites on the surface can increase the contact area with the electrolyte and promote the reduction process of electrolyte. Therefore, from both its own structure and chemical properties, it is highly feasible to use MoS_2_ as the CEs catalytic material of QDSCs.

In this study, chemical vapor deposition technology was applied to in‐situ deposit MoS_2_ nanoparticles on the surface of Co‐based MOF derivatives (Co,N─C materials) to obtain Co,N─C@MoS_2_ composite. Subsequently, the Co,N─C@MoS_2_ composite was mixed with binders to prepare a slurry, which was printed onto the surface of Ti mesh using screen printing technology to obtain Ti mesh supported Co,N─C@MoS_2_ composite CEs (Co,N─C@MoS_2_/Ti) for the assembly of QDSCs. This work utilizes the synergistic effect of two materials to obtain ideal CEs with excellent catalytic activity, conductivity, hydrophilicity, charge transfer rate, and stability. These factors contribute to the reduction of electrolyte, reducing the charge recombination, and further improving the photovoltaic performance of QDSCs. The QDSCs assembled based on Zn─Cu─In─S─Se (ZCISSe) QDs and Co,N─C@MoS_2_/Ti CEs achieved a PCE of 16.39% (*J*_sc_ = 27.26 mA cm^−2^, *V*_oc_ = 0.818 V, FF = 0.735). This work has contributed to promote the rapid improvement of QDSCs in PCE, and accelerated the development process of QDSCs in the photovoltaic fields.

## 2 Results and Discussion

### 2.1 Preparation and Characterization of Co,N─C Materials

**Figure 1:**
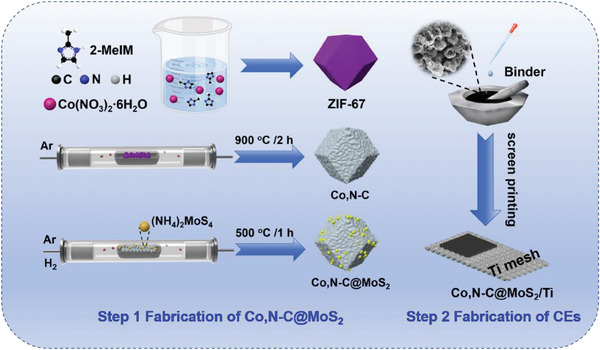
Schematic demonstration of the preparation of Co,N─C, Co,N─C@MoS_2_, and Co,N─C@MoS_2_/Ti CEs.

Co,N─C materials are prepared simply through the ZIF‐67 pyrolysis. The prepared process is illustrated in **Figure** **1**. During the annealing process, the organic ligands of MOF structure are carbonized to form amorphous carbon, while metal Co is reduced to a metallic elemental. It should be noted that its crystallization performance and carbonization processes are affected by the temperature. When the temperature is low, carbonization is incomplete and there are still existing metal coordination ions. Correspondingly, if the temperature is too high, it will cause the metal‐ligand skeleton to easily collapse and cause structural changes. Therefore, after preparing ZIF‐67, the temperature for carbonization into Co,N─C materials was optimized, and the XRD characterization corresponding to different materials synthesized at different temperatures is shown in Figure S1a (Supporting Information). The diffraction peaks at 44.2°, 51.5°, and 76.0° positions increase in intensity with increasing temperature, which corresponds to the Co (111), Co (200), and Co (220) crystal planes, respectively.^[^ ^35^ ^]^ The results confirm that the metal‐organic composite generated in the experiment is a cobalt phase, and the results corresponding to these crystal planes in the graph are consistent with the standard card of Co element. For Figure S1a (Supporting Information), there is also a weak diffraction peak at 26.20°, it corresponds to the diffraction peak of C (002) crystal plane, indicating that the cobalt ions are pyrolyzed into cobalt elemental substance after the annealing treatment. During the high‐temperature annealing process of materials, the metal components are decomposed into metal oxides or zero‐valent metals, and some elements in organic ligands, such as nitrogen, exist in the form of nitrogen compounds. And there will also be some forms of C converted into organic ligands present in the composite. Therefore, the ZIF‐67 material was pyrolyzed into Co,N─C materials after annealing.

In QDSCs, carbon‐based materials are often used as CEs catalytic materials, which is attributed to the good catalytic effects on polysulfide electrolytes. Therefore, the presence of excess Co elements is not conducive to reflecting their catalytic performance. In order to remove the free Co element from the Co,N─C material, the prepared composite was treated with 10 wt% H_2_SO_4_ aqueous solution. Figure S1b (Supporting Information) illustrates the XRD patterns of Co,N─C material after H_2_SO_4_ treatment. It can be seen that the diffraction peak of C (002) is significantly enhanced after H_2_SO_4_ treatment. With the temperature increases, the peak intensity becomes stronger, and the three crystal peaks of Co are significantly weakened, indicating that Co elemental in the composite can be removed effectively by H_2_SO_4_ treatment. According to the XRD characterization results, there are both obvious C and Co peaks at the annealing temperature of 900 °C, resulting in the formation of Co,N─C composite. However, if the temperature is below 900 °C, the diffraction peak of carbon is not obvious. On the contrary, when the temperature is beyond 900 °C, a relatively weak C (100) diffraction peak can be observed near the Co (111) crystal plane, leading to the complex composition of Co element in composite. Therefore, in the future, if there is no special mention, the annealing temperature for preparing Co,N─C is set to 900 °C.

### 2.2 Preparation and Characterization of Co,N─C@MoS_2_ Materials

The preparation of Co,N─C@MoS_2_ composite was carried out by in situ vapor deposition of MoS_2_ nanoparticles on the surface of Co,N─C.^[^ ^36^ ^]^ The detailed operation process is as follows, the obtained Co,N─C powders were mixed with (NH_4_)_2_MoS_4_/DMF solution (0.05 m), subsequently the mixture was heated to evaporate DMF completely, and then transferred to a tube furnace. The pyrolysis process of (NH_4_)_2_MoS_4_ in an argon atmosphere, MoS_2_ is deposited on the surface of Co,N─C. The schematic diagram of the preparation process is shown in Figure 1.

**Figure 2:**
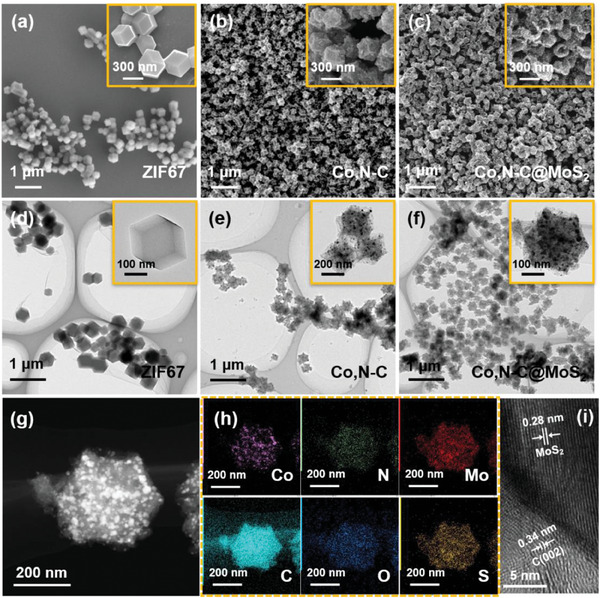
SEM and TEM images of a,d) ZIF 67, b,e) Co,N─C, and c,f) Co,N─C@MoS_2_ materials; g) TEM and h) the corresponding EDS‐mapping of Co,N─C@MoS_2_ composite; i) HR‐TEM image of Co,N─C@MoS_2_.

The synthesized sample was dispersed with ethanol, applied dropwise onto a silicon wafer for scanning electron microscopy determination, and added dropwise onto a copper mesh for transmission electron microscopy testing to observe its morphology. The SEM images of ZIF‐67, Co,N─C, and Co,N─C@MoS_2_ are shown in **Figure** **2**a,c. It can be seen that ZIF‐67 has a smooth surface and regular dodecahedral structure, and its organic framework structure has contracted to some extent after annealing. After subsequent treatment with (NH_4_)_2_MoS_4_, the presence of deposited MoS_2_ nanoparticles can be clearly observed on its surface. In addition, the enlarged SEM image in Figure 2c shows that MoS_2_ nanoparticles were successfully uniformly coated on the surface of Co,N─C materials. At the same time, the prepared materials were characterized by TEM, and the results are shown in Figure 2d–f, which are consistent with the SEM morphology characterization. In order to analyze the differences between Co,N─C and Co,N─C@MoS_2_ detail, TEM images at high magnifications were examined, and the results are shown in Figure S2a,b (Supporting Information). Compared with Co,N─C, Co,N─C@MoS_2_ exhibits a distinct MoS_2_ layered structure and mussy lattice fringes, which may be due to the stacking effect of MoS_2_ material in Co,N─C material. Therefore, it can further prove the successful synthesis of Co,N─C@MoS_2_ composite. Moreover, energy dispersive X‐ray spectroscopy (EDS) was conducted on the Co,N─C@MoS_2_ composite (Figure 2g,h), which also simply and intuitively provides the elemental content information of Co,N─C@MoS_2_. The results show that Mo and S elements are uniformly distributed around C element, indicating the successful preparation of Co,N─C@MoS_2_ composite. As a comparison, ZIF‐67 derived Co,N─C materials were also investigated by elemental energy spectrum analysis, and the results are shown in Figure S3 (Supporting Information). It can be seen that the number of nanoparticles on the surface of the framework has significantly decreased, and no signals of Mo and S elements have been found, indicating that the deposition of MoS_2_ is indeed caused by the decomposition of (NH_4_)_2_MoS_4_. In addition, the high‐resolution TEM (HR‐TEM) image of Co,N─C@MoS_2_ composite is also shown in Figure 2i, illustrating two different lattice spacings with 0.34 nm corresponding to the C (002) crystal plane spacing and 0.28 nm corresponding to the crystal plane spacing of MoS_2_ nanoparticles respectively.^[^ ^25^, ^35^ ^]^ These results further demonstrate that the Co,N─C@MoS_2_ composite can be achieved by in‐situ deposition of MoS_2_ nanoparticles on the surface of Co,N─C catalysts.

**Figure 3:**
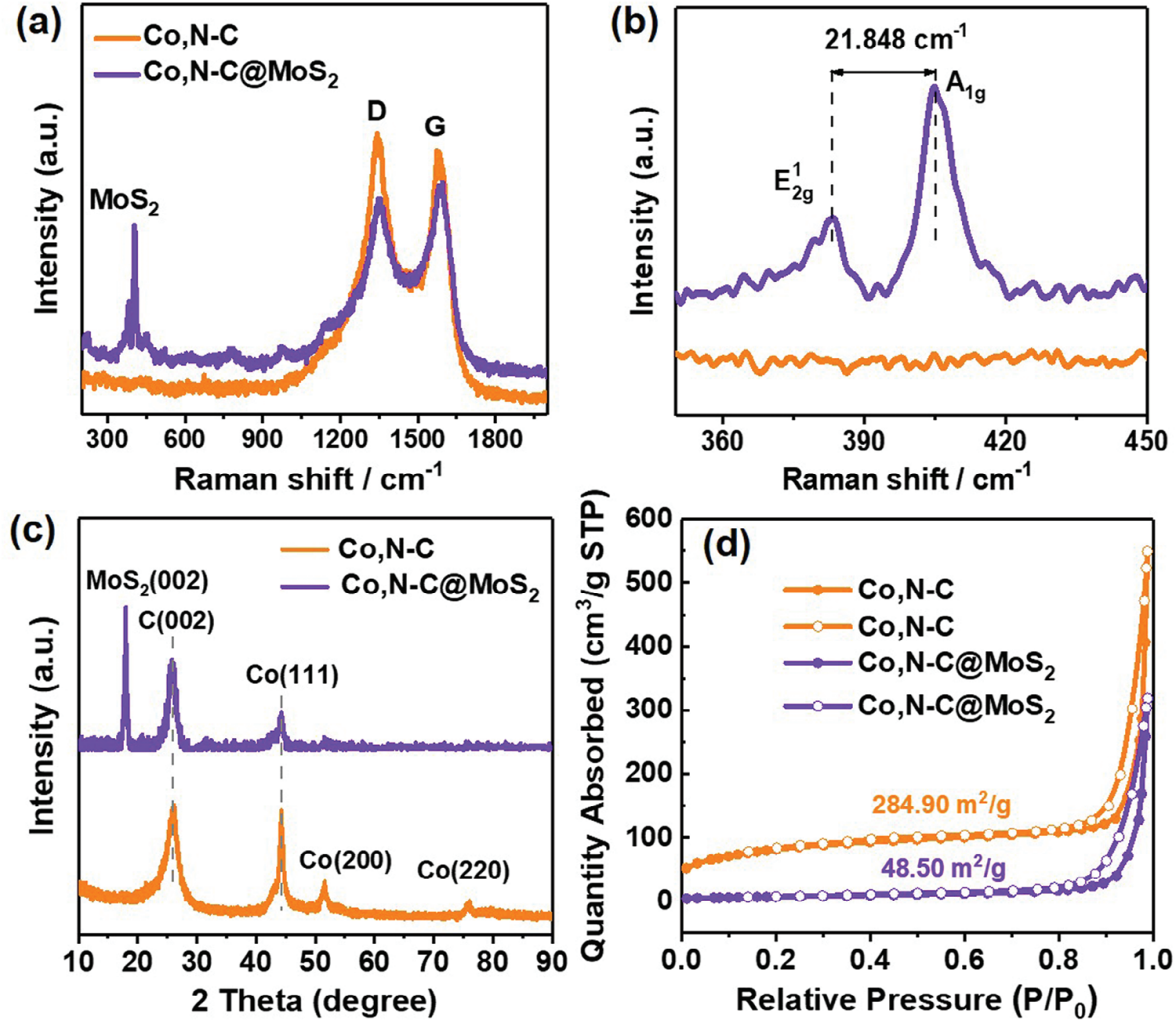
a) Raman scanning patterns of Co,N─C and Co,N─C@MoS_2_ samples; b) enlarged Raman curves; c) the XRD patterns and d) N_2_ adsorption–desorption curves of Co,N─C and Co,N─C@MoS_2_ samples.

**Figure** **3** shows the structural characterization results of Co,N─C material and Co,N─C@MoS_2_ composite. Raman spectroscopy is a sensitive method that can clearly identify the structure of MoS_2_ and carbon materials. Figure 3a shows the Raman spectra of Co,N─C material and Co,N─C@MoS_2_ composite. It can be found that there are two obvious Raman peaks at ≈1340 and 1585 cm^−1^ for the two kinds of materials. They correspond to the D‐band and G‐band of carbon materials respectively.^[^ ^37^ ^]^ Due to the presence of cobalt in the form of Co atoms in Co,N─C@MoS_2_ composite, there are no vibrations or rotational degrees of freedom for single atom molecules, so there will be no Raman characteristic peak. The presence of Raman peaks is attributed to the C elements from the carbonized ZIF‐ 67 materials by annealing treatment. Among them, the D‐peak mainly represents graphitic carbon structure with many defects or topological defects caused by heteroatom doping. The higher value illustrates the more defects in carbon atomic crystals in the material. By contrast, the G band deals with the movement of stretching fields between sp^2^ atoms, which is relatively less affected by temperature and other external environments. Moreover, the intensity ratio of the D‐band to the G‐band (*I*_D_/*I*_G_) was generally used to characterize the degree of graphitization of the material. It can be seen that the D‐band intensity decreased, and the intensity of G‐band increased for Co,N─C@MoS_2_, indicating that the introduction of MoS_2_ nanoparticles will be beneficial to reduce the defect of carbon materials. The reduction of defect states in composite materials mainly includes the following three aspects. i) MoS_2_ nanoparticles with layered structure may fill the defective sites in Co,N─C materials, resulting in a reduction of the original defect states. ii) A chemical bonds may be formed between MoS_2_ and Co,N─C materials, such as Co─S bond, which contribute to stabilize the material structure and reduce defects. iii) MoS_2_ is a material with a unique electronic structure. When it is combined with Co,N─C material, it may affect the electronic state of Co,N─C material and reduce the defect states. Compared with Co,N─C materials, Co,N─C@MoS_2_ composite exhibits Raman peaks of MoS_2_ crystals. The detailed results and enlarged Raman spectra are illustrated in Figure 3b. There are two distinct characteristic peaks at ≈380 and 404 cm^−1^ for Co,N─C@MoS_2_ sample, corresponding to the in‐plane Mo‐S phonon mode (*E*1 2 g) and the out‐of‐plane Mo‐S mode (A_1g_) of typical MoS_2_‐layered structure, respectively. It indicates that Co,N─C@MoS_2_ composite have been prepared successfully, and MoS_2_ nanoparticles illustrate a typical 2H phase.^[^ ^36^ ^]^ In addition, the structure of Co,N─C and Co,N─C@MoS_2_ samples were also determined by XRD. The powder XRD patterns of the two samples are shown in Figure 3c. The main diffraction peak of the Co,N─C@MoS_2_ composite at 2θ of 18^°^ exhibits 2H‐MoS_2_ (002) crystal planes, which are consistent with Raman characterization results.^[^ ^25^ ^]^ These results further demonstrated that the MoS_2_ materials were deposited on the surface of Co,N─C successfully.

Subsequently, N_2_ adsorption–desorption experiments were conducted to investigate the specific surface area of the obtained Co,N─C and Co,N─C@MoS_2_ materials. As shown in Figure 3d, a typical H3‐type hysteresis loop can be observed in both samples, indicating that Co,N─C and Co,N─C@MoS_2_ materials have a mesoporous structure. Meanwhile, in contrast to traditional Co,N─C materials with high surface areas (284.90 m^2^ g^−1^), Co,N─C@MoS_2_ illustrated relatively low Brunauer Emmett Teller values (48.50 m^2^ g^−1^). The results suggested that the introduction of MoS_2_ reduces the surface area of the obtained carbon materials, which is unfavorable for high activity performance of obtaining the CE catalysts. Due to the possibility of providing more active sites for materials with a larger specific surface area, the added amount of MoS_2_ was investigated in the subsequent preparation of the CEs.

**Figure 4:**
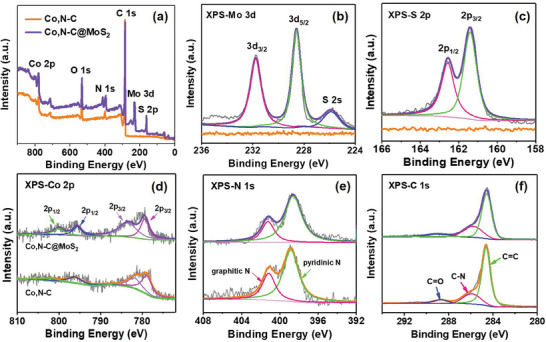
a) XPS full‐scale spectra of Co,N─C and Co,N─C@MoS_2_; High‐resolution XPS spectra of b) Mo 3d, c) S 2p, d) Co 2p, e) N 1s, and f) C 1s of Co,N─C and Co,N─C@MoS_2_ samples.

In addition, X‐ray photoelectron spectroscopy (XPS) measurements further investigate the elemental composition and chemical bonding state of materials for Co,N─C and Co,N─C@MoS_2_ samples, and XPS analyses further verify the five elements of Co, N, C, Mo, and S in nanocomposite shown in **Figure** **4**a. It should be noted that the presence of O element may be ascribed to the superficial oxidation of Co,N─C@MoS_2_ as a result of air contact. Moreover, a silicon wafer with SiO_2_ layer was used as the substrate for XPS testing, resulting in the higher signal intensity of O 1s compared to the others in both Co,N─C and Co,N─C@MoS_2_ materials. Both materials illustrate similar XPS peak positions for same elements, such Co, N, and C. However, Co,N─C@MoS_2_ composite exhibits the incremental Mo 3d peaks and S 2p peaks in comparison with that Co,N─C material.^[^ ^26^ ^]^ As shown in the high‐resolution XPS spectra of Figure 4b, the binding energies of Mo 3d_3/2_ and Mo 3d_5/2_ are 231.75 and 228.55 eV for 2H phase, which is attributed to Mo in Co,N─C@MoS_2_ composite. Similarly, the spectrum in Figure 4c has two distinct peaks at 162.6 eV for S 2p_1/2_ and 161.4 eV for S 2p_3/2_, indicating that sulfur element exists as S^2‐^ ions. Figure 4d displays the high‐resolution Co 2p core level XPS spectrum, which can be fitted into four peaks. Two obvious peaks at 796.75 and 779.15 eV separately correspond to Co 2p_1/2_ and Co 2p_3/2_ for Co^3+^; two peaks at 800.05 and 783.65 eV originate from Co^2+^ 2p_1/2_ and Co^2+^ 2p_3/2_, respectively. As for N 1s core level XPS spectrum (Figure 4e), two fitted peaks ≈398.85 and 401.25 eV may be assigned to pyridinic and graphitic N Species. What's more, the C 1s peaks at 284.7, 285.8, and 288.75 eV shown in Figure 4f can be assigned to carbon in the form of C═C, C─N, and C═O respectively. It is noted that the presence of C═O signal may be attributed to the oxygen species from the moisture absorbed on the material surfaces. Therefore, the results of XPS further proved the successful preparation of Co,N─C@MoS_2_ composite.

### 2.3 Photovoltaic Performances

**Figure 5:**
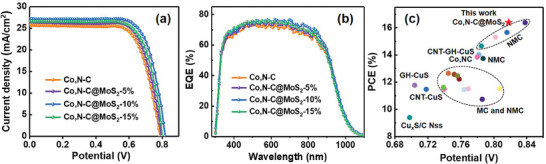
Photovoltaic performance characterizations of ZCISSe QDSCs based on Co,N─C/Ti CEs and Co,N─C@MoS_2_/Ti CEs. a) *J–V* curves; b) EQE curves; c) the comparison of QDSC performances based different CEs.

Typical sandwich‐type ZCISSe QDSCs were selected as a typical device to investigate the effect of different CEs since the highest PCE was derived from this type of solar cell. The photovoltaic performance, such as current density–voltage (*J–V*) curves, of QDSCs based on Co,N─C/Ti and Co,N─C@MoS_2_/Ti CEs under 1 full sun illumination are shown in **Figure** **5**a. Moreover, five parallel CEs under same conditions were prepared for the ZCISSe QDSCs to guarantee the reliability of the given data, and the corresponding photovoltaic performance was detected. The *J–V* curves and the average photovoltaic parameters (including short‐circuit, *J*_sc_; open‐circuit voltage, *V*_oc_; fill factor, FF, and PCE) for the devices under different conditions are shown in Figure S4 (Supporting Information) and **Table** **1**. In addition, the PCE values from *J–V* curves of QDSCs based on different CEs were summarized in Figure S5a (Supporting Information). For clarity, the dependence of the individual photovoltaic parameter together with the corresponding deviation value of cell devices with different MoS_2_ nanoparticle dopant ratios in the CEs are illustrated in Figure S5b,c (Supporting Information). From these data, it is found that *J*_sc_, *V*_oc_, and FF values increased systematically with an increase in the MoS_2_ amounts in CE catalysts and approached the highest PCE values when the MoS_2_ concentration was 10 wt%. When the MoS_2_ doping amount was beyond 10 wt%, the main parameters started to decrease gradually. Among them, the ZCISSe‐based QDSCs, when Co,N─C@MoS_2_‐5%, Co,N─C@MoS_2_‐10%, and Co,N─C@MoS_2_‐15% are used as the electrocatalyst in CEs, the devices illustrate champion PCE values of 15.38% (*J*_sc_ = 26.71 mA cm^−2^, *V*_oc_ = 0.792 V, FF = 0.727), 16.39% (*J*_sc_ = 27.26 mA cm^−2^, *V*_oc_ = 0.818 V, FF = 0.735) and 15.63% (*J*_sc_ = 26.72 mA cm^2^, *V*_oc_ = 0.808 V, FF = 0.724), which are 5.3%, 12.2%, and 7.1% higher than that of QDSCs based Co,N─C CEs with PCE of 14.60% (*J*_sc_ = 25.74 mA cm^−2^, *V*_oc_ = 0.792 V, FF = 0.716), respectively. It is highlighted that the obtained properties of QDSCs stayed at the same level as the best‐reported result for Ti mesh CEs. Compared with the PCE values reported by other literatures for different catalysts (Figure 5c), it is obvious that the QDSCs with Co,N─C@MoS_2_/Ti CEs feature a relatively high PCE and excellent properties. The representative photovoltaic parameters for QDSCs based on different CEs are summarized in Table S2 (Supporting Information). The improved performance is derived from the increase of *V*_oc_ and FF values, while the *J*_sc_ also has a slight enhancement. The results reflect the superior performance of CEs may be attributed to the introduction of MoS_2_ nanoparticles, which contributed to the enhancement of electrocatalytic and the reduction of polysulfide. However, compared to the decreased PCE can be observed for Co,N─C@MoS_2_‐15% CEs. The reason is mainly attributed to the excessive presence of MoS_2_, which reduces the content of carbon materials in the composite catalysts.

**Table 1:** Individual and average photovoltaic parameters of ZCISSe QDSCs based on different CEs under the illumination of 1 full sun intensity (AM 1.5G, 100 mW cm^−2^).

CEs	*J*_sc_ [mA cm^−2^]	*V*_oc_ [V]	FF	PCE [%]
Co,N─C/Ti	25.86	0.784	0.716	14.52
	25.77	0.786	0.719	14.56
	25.74	0.792	0.716	14.60
	25.69	0.779	0.714	14.29
	26.03	0.776	0.721	14.56
**average**	**25.82 ± 0.13**	**0.783 ± 0.006**	**0.717 ± 0.003**	**14.51 ± 0.12**
Co,N─C@ MoS_2_‐5%/Ti	26.52	0.795	0.725	15.29
	26.71	0.792	0.727	15.38
	26.49	0.796	0.728	15.35
	26.35	0.791	0.722	15.05
	26.42	0.795	0.724	15.21
**average**	**26.50 ± 0.13**	**0.793 ± 0.002**	**0.725 ± 0.002**	**15.25 ± 0.13**
Co,N─C@ MoS_2_‐10%/Ti	27.26	0.818	0.735	16.39
	27.08	0.808	0.740	16.19
	26.86	0.815	0.734	16.07
	27.25	0.812	0.737	16.31
	26.94	0.820	0.738	16.30
**average**	**27.08 ± 0.18**	**0.814 ± 0.005**	**0.737 ± 0.002**	**16.25 ± 0.12**
Co,N─C@ MoS_2_‐15%/Ti	26.63	0.800	0.729	15.53
	26.72	0.808	0.724	15.63
	26.31	0.804	0.731	15.46
	26.38	0.798	0.723	15.22
	26.55	0.795	0.728	15.37
**average**	**26.52 ± 0.17**	**0.801 ± 0.005**	**0.727 ± 0.003**	**15.44 ± 0.16**

As an important component of QDSCs, the CEs play two main roles, one hand is to accept the external circuit electrons and efficiently transfer them, and the other hand can be catalyzed to the reduction of electrolyte between the CEs and electrolyte interface. A kind of ideal CE material serving as a substrate in transferring electrons from the external circuit to the polysulfide aqueous electrolyte. Therefore, the hydrophilicity of catalysts plays an important role in the interactions between heterogeneous catalysts and reactants, consequently directly affecting their activity. To investigate the wettability of Co,N─C and Co,N─C@MoS_2_ composite to water, the two kinds of materials were dispersed into a mixture of methyl acetate and water solution. The results shown in Figure S6a (Supporting Information) illustrated that the composite can be well dispersed in the water phase, indicating that the presence of MoS_2_ has no influence on the hydrophilicity of Co,N─C materials. Moreover, contact angle tests of CEs were also done, and the corresponding photographs are illustrated in Figure S6b (Supporting Information). It can be found that the contact angle of Co,N─C/Ti CEs is ≈25.9°, showing good hydrophilicity. For Co,N─C@MoS_2_/Ti CEs, the hydrophilicity of the corresponding sample was enhanced with the contact angle reduced to 21.6° (Figure S6c, Supporting Information). The enhanced hydrophilicity of Co,N─C@MoS_2_/Ti CEs was ascribed to the existence of MoS_2_ that induces the permanent polarization between graphitic‐N and adjacent C and thus facilitates the interaction between N species and nucleophilic water molecules.

It should be noted that the measured result of *J*_sc_ values for different materials are also reflected by EQE characterization, and the data are shown in Figure 5b. The curves of QDSCs based on the different CEs illustrate a similar trend in the whole photocurrent response range, and the EQE values of Co,N─C@MoS_2_‐10% CEs show a slight increase within the response range of 400–800 nm. Among them, the corresponding integral current values according to the EQE spectra improve from 25.51 to 26.94 mA cm^−2^ for ZCISSe QDSCs based on the Co,N─C/Ti CEs and Co,N─C@MoS_2_‐10%/Ti CEs, respectively. The results are consistent with the *J*_sc_ values in the *J–V* detection (Table S1, Supporting Information).

### 2.4 Electrochemical Characterization

**Figure 6:**
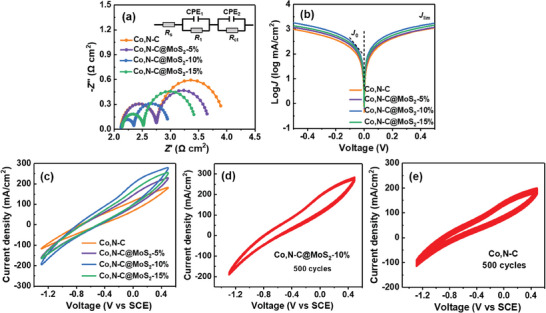
Electrochemical characterization of ZCISSe QDSCs based on various CEs. a) Nyquist plots (inset: simulation circuit used for analyzing the EIS data); b)Tafel polarization curves and c) CVs based on different CE catalysts; Consecutive CV measurement for 500 cycles for d) Co,N─C@MoS_2_‐10%/Ti and e) Co,N─C/Ti CEs at a scan rate of 100 mV s^−1^ in the media of polysulfide redox electrolyte.

In order to investigate the mechanism for the improved photovoltaic performance of the Co,N─C@MoS_2_/Ti CE‐based ZCISSe QDSCs, an electrochemical impedance spectroscopy (EIS) measure was conducted on Co,N─C@MoS_2_/Ti CE fabricated by the printed of Co,N─C@MoS_2_ composite on Ti mesh substrates.^[^ ^38^ ^]^ Typically, two identical CEs were used to form a virtual cell unit and then dipped into the polysulfide electrolyte to maintain a distance of 1 cm between the two CEs. This cell structure can exclude interference from other components in a complete cell device. The Nyquist plots obtained from this test are shown in **Figure** **6**a, and the fitting parameters shown in **Table** **2** are extracted with the use of a standard equivalent circuit (inset of Figure 6a). In Nyquist plots, the intercept of the horizontal axis denotes the series resistance (*R*_s_) of the CEs. Two semicircles can be observed in the Nyquist plots of both CEs. The first semicircle at high frequencies is generally considered to be related to the resistance (*R*_1_) of the solid‐solid contact between the substrate and the carbon material. The second semicircle at the lower frequency corresponds to the charge‐transfer resistance (*R*_ct_) at the solid–liquid phase interface between the electrolyte and the carbon material. It could be seen that all CEs show similar *R*_s_ values as the previous report due to the superior conductivity of Ti mesh substrate. Differently, the *R*_ct_ values of the Co,N─C@MoS_2_/Ti CEs are substantially lower than that of Co,N─C/Ti CE (1.532 Ω cm^2^), and show the lowest value at the MoS_2_ concentration of 10%. The lower *R*_ct_ values correspond to better catalytic reduction activity and a higher FF value in QDSCs, which may be ascribed to the increased catalytic active sites from MoS_2_ nanoparticles. In addition, the increase of catalytic active sites will improve the reduction ability of *S*_n_^2−^ to *S* ^2−^, which is propitious to reduce the reaction barrier of the reduction of the polysulfide electrolyte. When the MoS_2_ concentration was increased to 15%, the corresponding *R*_ct_ value from EIS parameters decreased on the contrary. This reason is mainly attributed to the decrease in the conductivity of composite.

**Table 2:** Parameters from EIS for different types of CEs.

CEs	*R*_s_ [Ω cm^2^]	*R*_1_ [Ω cm^2^]	*R*_ct_ [Ω cm^2^]
Co,N─C/Ti	2.128	0.380	1.532
Co,N─C@MoS_2_‐5%/Ti	2.147	0.368	1.121
Co,N─C@MoS_2_‐10%/Ti	2.123	0.207	0.558
Co,N─C@MoS_2_‐15%/Ti	2.145	0.276	0.834

Except for EIS testing, Tafel polarization curves were also characterized to further investigate the catalytic performance of different CEs, and the results are shown in Figure 6b. The exchange current density (*J*_0_) increases with the enhancement of MoS_2_ concentration in the composite, and obtains the best properties at MoS_2_ concentration of 10%, illustrating that an obvious greater than that for Co,N─C materials. In addition, the *J*_lim_ values for Co,N─C@MoS_2_/Ti CEs are also higher than that of Co,N─C/Ti CE, implying the higher diffusion of polysulfide redox in the electrolyte. To further study the electrocatalytic activity of the CEs toward the reduction of polysulfide electrolyte, cyclic voltammetry (CV) measurement was performed at a scan rate of 100 mV s^−1^. It is well‐acknowledged that a higher current corresponds to a better electrocatalytic performance of CEs in CV curves. As illustrated in Figure 6c, Co,N─C@MoS_2_‐10%/Ti CE exhibits the highest current density among the other CEs. The introduction of MoS_2_ can effectively promote the reduction process of the electrolyte, thereby enhancing the electrocatalytic activity of electrode. The result is conformable with the variation of *R*_ct_ value obtained in EIS measurement, proving that Co,N─C@MoS_2_/Ti CEs exhibit better catalytic activity. It should be noted that the peak shape and intensity of CV are related to the catalytic activity of CEs, regardless of the material type. Generally speaking, the CV curves for different CEs display two peaks. The negative current corresponds to the reduction reaction of S_n_^2−^ to S^2−^, while the other one (positive current) is assigned to the oxidation of S^2−^ in electrolyte.

Besides, the high stability of the CEs is a significant factor for the further practical application in QDSCs. Here, the stabilities of CEs were investigated through the continuously electrochemical CV scan, and the results are shown in Figure 6d. It can be seen that there were nearly no significant changes in the curve shape after 500 cycles for Co,N─C@MoS_2_‐10%/Ti CEs under the continuous scan, whereas a slight decrement was observed in the Co,N─C@/Ti CEs. The stability mechanism may be ascribed to the monolayer of electrolyte molecules that was adsorbed on both sides of the MoS_2_ nanoparticle and the hydrophilicity advantage of MoS_2_ materials.

### 2.5 Long‐Term Stability

**Figure 7:**
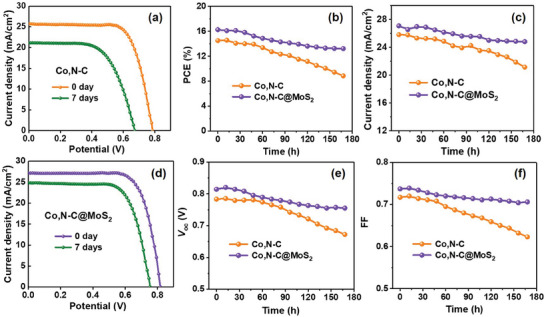
Stability of *J–V* curves of QDSCs at 0 day and after 7 days: a) with Co,N─C/Ti CEs; d) Co,N─C@MoS_2_/Ti CEs. Dependence of photovoltaic parameters of QDSCs at different times: b) PCE; c) *J*_sc_; e) *V*_oc_; f) FF.

The long‐term stability of QDSCs is crucial for practical applications. Herein, the stability of the device is evaluated by continuously testing the photovoltaic performance for 15 days, and the detailed results are shown in **Figure** **7**. Figure 7a,d displays the *J–V* curves of QDSCs with Co,N─C/Ti and Co,N─C@MoS_2_/Ti CEs respectively. The specific corresponding photovoltaic parameters under different times are shown in Figure 7b,c,e,f. QDSCs were characterized under the illumination of 1 full sun intensity (AM 1.5, 100 mW cm^−2^) at room temperature. After each use, the QDSC was stored at ambient dark conditions. As illustrated in Figure 7, the stability of QDSCs‐based Co,N─C@MoS_2_/Ti CEs was slightly superior compared to that of the Co,N─C/Ti CE. All the photovoltaic parameters of QDSCs based both CEs decrease as time increases. The rate of decrease is higher for QDSCs with Co,N─C@MoS_2_/Ti CEs than QDSCs with Co,N─C/Ti CE. Among them, the crucial PCE values decreased by ≈40% and 19% for Co,N─C/Ti and Co,N─C@MoS_2_/Ti CEs respectively after 7 days, compared to their initial PCE, which exhibited a relatively acceptable stability. Specifically, after 7 days since construction, other photovoltaic parameters such as *V*_oc_ decreases from 0.783 to 0.672 V with a decline of 14%, *J*_sc_ decreases from 25.82 to 21.15 mA cm^−2^ with a decline of 18%, and FF decreases from 0.717 to 0.623 with a decline of 13% for QDSCs with Co,N─C/Ti CEs. For QDSCs based Co,N─C@MoS_2_/Ti CEs, *V*_oc_ decreases from 0.814 to 0.755 V with a decline of 7%, *J*_sc_ decreases from 27.08 to 24.81 mA cm^−2^ with a decline of 8%, and FF decreases from 0.737 to 0.706 with a decline of 4%. This difference in the long‐term stability of QDSCs is mainly attributed to better electrocatalytic activity of Co,N─C@MoS_2_/Ti CEs as compared to Co,N─C/Ti CEs. In addition, the electrolyte can be easily adsorbed on the surface of MoS_2_ nanoparticles, leading to high stability in QDSC devices.

Actually, it is worth noting that the individual Co,N─C@MoS_2_/Ti CE can still maintain good catalytic activity even after being left at room temperature in the air for more than 2 months. The main reason for the decrease in stability is ascribed to the declining performance of photoanode, which leads to a deterioration in the stability of the device performance. In addition, the SEM and XRD characterizations of Co,N─C@MoS_2_ composite after the 7‐day stability test were measured to understand the material degradation mechanisms. The results shown in Figure S7 (Supporting Information) suggest that no significant change for SEM except for the residual electrolyte modification on the material surface. According to XRD data analysis, there was also no change in the crystal structure after stability testing, indicating excellent stability of Co,N─C@MoS_2_ composite.

### 2.6 Density Functional Theory (DFT) Calculations

**Figure 8:**
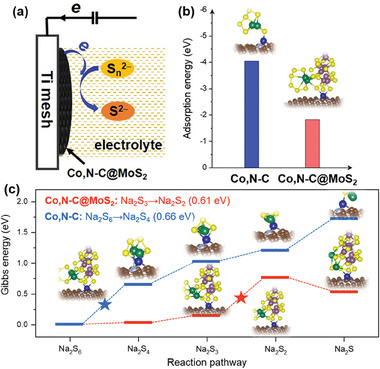
a) Schematic of interaction mechanism between Co,N─C@MoS_2_ and polysulfide electrolytes; b) Adsorption energy of Na_2_S_6_ on the surface of Co,N─C and Co,N─C@MoS_2_; c) Gibbs free energy of each elementary reaction from Na_2_S_6_ to Na_2_S on the Co,N─C and Co,N─C@MoS_2_ surface.

As illustrated in **Figure** **8**a, the role of CE is to collect the photogenerated electrons from the external circuit and catalyze the reduction of Na_2_S_n_ to Na_2_S at the interface of CE/electrolyte. To further verify the catalytic performance of Co,N─C@MoS_2_ composite in QDSCs, density functional theory (DFT) calculations were applied to investigate the reduction process of polysulfides occurring on the surface of CE. All the theoretical calculations were conducted employing the VASP code, utilizing the Perdew‐Burke‐Ernzerhof (PBE) functional to describe exchange‐correlation energy.^[^ ^39^, ^40^, ^41^ ^]^

According to the experimental results, a Co and N co‐doped carbon was first selected as the substrate (Co,N─C), and then loaded MoS_2_ cluster on top to build a composite structure of Co,N─C@MoS_2_. Note that the formation of the MoS_2_/Co, N─C interface effectively modulates the electronic structure of the counter electrode, thereby promoting rapid charge transfer at the interface and improving the utilization efficiency of photogenerated electrons (Figure S8, Supporting Information). Then, Na_2_S_6_ was chosen as the initial reactant throughout the entire reduction process, as it represents the highest valence oxidation species that may exist in the polysulfide electrolyte. To explore the mechanism of Na_2_S_6_ reduction, the conversion of Na_2_S_n_ (n = 6, 4, 3, 2, and 1) intermediates was investigated on both the Co,N─C and Co,N─C@MoS_2_ surfaces. The cut‐off energy was set at 520 eV, and structural relaxation was carried out with energy convergence criteria of 10^−5^ eV atom^−1^ and force convergence criteria of 0.01 eV Å^−1^, respectively. A Monkhorst–Pack grid of 3 × 3 × 1 k‐points was employed to sample the Brillouin zone. The vdW‐DF2 method was employed to account for the long‐range van der Waals (vdW) interactions within all structures.^[^ ^42^, ^43^ ^]^ As expected, the Na_2_S_6_ can adsorb on the catalytic surfaces of Co,N─C and Co,N─C@MoS_2_ spontaneously, showing adsorption energies of −4.0 and −1.8 eV respectively (Figure 8b). The charge density difference plots reveal that the adsorption of Na_2_S_6_ induces obvious charge transfer on both the Co,N─C and Co,N─C@MoS_2_ surfaces (Figure S9, Supporting Information). This process effectively facilitates charge transfer from the surfaces to Na_2_S_6_, thereby activating the reactant. However, the distinct areas of charge accumulation and depletion suggest that the catalytic mechanisms on the Co,N─C and Co,N─C@MoS_2_ surfaces may differ. Specifically, due to the high catalytic activity of Co sites of Co,N─C, Na_2_S_6_ tends to bond with Co,N─C by forming stronger Co─S bond, whereas the adsorption of Na_2_S_6_ on the Co,N─C@MoS_2_ surface preferably occurs through the moderate bonding of Na atom with the S atoms of MoS_2_ cluster. Moreover, possible reduction pathways of Na_2_S_6_ on Co,N─C, and Co,N─C@MoS_2_ are described in Figure 8c, and the corresponding Gibbs energy (*∆*G) is summarized in **Table** **3**. It can be seen that the first reduction step of Na_2_S_6_ → Na_2_S_4_ is more feasible on Co,N─C@MoS_2_ (0.03 eV) than Co,N─C surface (0.66 eV), which should be ascribed to the adsorption configuration of Na_2_S_6_ on Co,N─C@MoS_2_. This moderate binding mode is advantageous not only for the activation of Na_2_S_6_ but also for subsequent conversion reactions of polysulfides. In addition, the rate‐determining step throughout the polysulfides reduction is also different, the Co,N─C surface is hindered by the reduction process from Na_2_S_6_ to Na_2_S_4_ (0.66 eV), while the conversion reaction from Na_2_S_3_ to Na_2_S_2_ is the limiting step on Co,N─C@MoS_2_ with an energy barrier of 0.62 eV. Thus, the introduction of MoS_2_ nanocluster can efficiently control the catalytic mechanism of Na_2_S_6_ reduction by optimizing the adsorption configurations of polysulfide intermediates, thereby reducing the reaction energy barrier of the whole reduction pathway.

**Table 3:** The standard Gibbs free energy of CEs catalyst toward the reduction reaction of polysulfide.

CEs catalyst	Reaction	Reaction of *∆*G [eV]
Co,N─C	Na_2_S_2_ → Na_2_S	0.50
	Na_2_S_3_ → Na_2_S_2_	0.20
	Na_2_S_4_ → Na_2_S_3_	0.36
	Na_2_S_6_ → Na_2_S_4_	0.66
	Na_2_S_6_ adsorption	−4.04
Co,N─C@MoS_2_	Na_2_S_2_ → Na_2_S	−0.23
	Na_2_S_3_ → Na_2_S_2_	0.62
	Na_2_S_4_ → Na_2_S_3_	0.12
	Na_2_S_6_ → Na_2_S_4_	0.03
	Na_2_S_6_ adsorption	−1.83

The possible reason can be attributed to the presence of S atom in MoS_2_ compound. It may feature a binding effect on intermediate polysulfides, which is similar to the stabilizers to steady the combined state. This may be ascribed to S in MoS_2_ coordinates with Na element, and the corresponding Na─S bond length is similar to Na─S in Na_2_S_4_. It should be noted that the reduction process from Na_2_S_3_ to Na_2_S_2_ is difficult to occur on the Co,N─C@MoS_2_ substrate. However, the *∆*G value of the reduction process from Na_2_S_2_ to Na_2_S was less than 0, indicating that the reduction reaction of polysulfides occurring on the Co,N─C@MoS_2_ surface is more thorough, which is favorable to the whole reduction pathway. Therefore, the DFT results exhibited that Co,N─C@MoS_2_ possessed higher catalytic activity in the reduction of polysulfides compared to Co,N─C materials, which is consistent with the electrochemical characterization conclusions.

## 3 Conclusion

In this work, in situ chemical vapor deposition of MoS_2_ nanoparticles on the surface of Co,N─C materials, as increasing active sites, affords highly electrocatalytic performance of Co,N─C@MoS_2_ composite. A titanium mesh substrate was selected to load the materials to fabricate Co,N─C@MoS_2_/Ti CEs. The morphology and structure of Co,N─C@MoS_2_/Ti electrodes were characterized carefully, including XRD, XPS, SEM, and electrochemical tests. It was found that the Co,N─C@MoS_2_/Ti illustrated relatively excellent electrocatalytic activity toward the reduction of polysulfide electrolyte compared to the Co,N─C/Ti CEs. Meanwhile, the prepared CEs indicate good stability under the continuous cyclic voltammetry scan. The assembled ZCISSe QDSCs based on Co,N─C@MoS_2_‐10%/Ti CEs reveal superiorly photovoltaic performance, which can be attributed to the exterior active sites of MoS_2_ nanoparticles to help reduce the series impedance and the improved electronic transmission in QDSCs. Moreover, DFT results indicated that the reduction step of Na_2_S_6_ → Na_2_S_4_ is more feasible on Co,N─C@MoS_2_ (0.03 eV) than Co,N─C surface (0.66 eV). With the Co,N─C@MoS_2_‐10%/Ti CEs, the highest PCE of 16.39% (*J*_sc_ = 27.26 mA cm^−2^, *V*_oc_ = 0.818 V, FF = 0.735) was achieved on ZCISe QDSCs, resulting in an approximate 12% increase in the PCE of reference CEs. These research results provide a new strategy for the design of high catalytic activity electrodes, and show the promising application of Co,N─C@MoS_2_ composite in QDSCs.

## 4 Experimental Section

### Chemicals

2‐Methylimidazole (2‐MeIM, 98%) and cobaltous nitrate hexahydrate (Co(NO_3_)_2_·6H_2_O, 99%) were purchased from Innochem Co. Ltd. Indium acetate (In(OAc)_3_, 99.99%), terpineol, and elemental selenium (200 mesh, 99.99%) were obtained from Sigma‐Aldrich. Mercaptopropionic acid (MPA, 97%) and copper iodide (CuI, 99.998%) were obtained from Alfa Aesar. 1‐octadecene (ODE, 90%) was purchased from *J*&*K*. Oleylamine (OAm, 95%) was purchased from ACROS. Sulfur powder (99.99%), zinc acetate (Zn(OAc)_2_·2H_2_O, 99.99%), sodium sulfide (Na_2_S·9H_2_O, 99.99%), and poly(vinylpyrrolidone) with 8000 molecular weight were obtained from Aladdin, China. Diphenylphosphine (DPP, 98%) and ammonium tetrathiomolybdate were obtained from Adamas Reagent Co. Ltd. (Shanghai, China).

### Preparation of ZIF‐67 Nanocrystals

ZIF‐67 was synthesized in the methanol phase: 8 mmol of Co(NO_3_)_2_·6H_2_O was dissolved in 100 mL of methanol and quickly poured into 100 mL of methanol solution containing 32 mmol of 2‐MeIM. After stirring at room temperature for 15 min to mix thoroughly, the solution was incubated for 24 h. The purple ZIF‐67 crystals were obtained by centrifugation, washed with methanol several times until clear, and finally dried at 80 °C for 12 h.

### Preparation of Co,N─C

The dried ZIF‐67 was placed in a tube furnace and carbonized through high‐temperature pyrolysis at Ar atmosphere. After 4 h of activation at 200 °C, the crystal undergoes pyrolysis at 800 °C, and the heating rate was controlled to be 5 °C min^−1^. Then the black product was immersed in a 10 wt% H_2_SO_4_ solution, ultrasonicated for 20 min, and continuously stirred at 80 °C for 6 h. The product was centrifuged, washed several times alternately with DI water and ethanol, and finally dried at 60 °C for 12 h.

### Preparation of Co,N─C@MoS_2_ and Co,N─C@MoS_2_/Ti CEs

To prepare Co,N─C@MoS_2_‐5%, Co,N─C@MoS_2_‐10%, and Co,N─C@MoS_2_‐15% samples, 3, 6, and 9 mL of 0.05 m DMF solutions of ammonium tetrathiomolybdate ((NH_4_)_2_MoS_4_) were mixed with 100‐mg Co,N─C powder respectively. The mixture was stirred for 12 h and then dried. The above product was packed in a crucible calcined at 500 °C for 1 h with a ramp rate of 10 °C min^−1^ in an atmosphere of 20 sccm H_2_ and 80 sccm Ar in a tube furnace. Generally speaking, it is rational to increase the thermolysis temperature to obtain high‐quality MoS_2_ nanocrystals in an N_2_ environment. However, the conversion of (NH_4_)_2_MoS_4_ to MoS_2_ was lowered to ≈500 °C in the presence of H_2_ gas, as described in Equation 1, which is ascribed to the MoS_2_ decomposes in H_2_ when the temperature is higher than 500 °C.

(1) $${\left(NH_{4}\right)}_{2}\mathrm{Mo}S_{4}+H_{2}\to 2NH_{3}+2H_{2}S+\mathrm{Mo}S_{2}$$

Consequently, 500 °C was set as the calcination temperature for the preparation of Co,N─C@MoS_2_ sample. However, it should be noted that since the specific conversion rate of (NH_4_)_2_MoS_4_ is unknown, the amount of MoS_2_ in the Co,N─C@MoS_2_ composite material was calculated by the difference in mass before and after the reaction.

### Fabrication of Solar Cells

TiO_2_ mesoporous films and ZCISSe QDs were prepared based on previous reports.^[^ ^10^ ^]^ Briefly, a Zn(OAc)_2_ stock solution was first prepared by dissolving Zn(OAc)_2_·2H_2_O (1.0 mmol) in a mixture of OAm (1 mL) and ODE (9 mL) at 130 °C under N_2_ atmosphere. Se precursor was prepared by dissolving Se powder (0.5 mmol) in a mixture of DPP (0.5 mL) and OAm (0.5 mL) under ultrasonication at room temperature. The S precursor (1.0 m) was prepared by dissolving S powder in DPP. For the synthesis of ZCISSe QDs, In(OAc)_3_ (0.2 mmol), CuI (0.14 mmol), Zn(OAc)_2_ (0.08 mmol), and OAm (10.0 mL) were loaded in a flask. The system was heated to 180 °C under N_2_ atmosphere, and 1.0 mL S/Se (6:4) mixture was quickly injected into the mixture, and then the system was heated to 220 °C and kept the reaction for 8 min. The obtained OAm ligand‐capped ZCISSe QDs were purified by centrifugation with the addition of ethanol.

The obtained ZCISSe QDs solution (50 µL) was dropped onto the TiO_2_ film and then stayed for 2 h at 60 °C. After QDs loading, ZnS passivation layer was coated on the surface of QDs by immersing alternately for 5 cycles in 0.1 m Zn(OAc)_2_ methanol solution and 0.1 m Na_2_S aqueous solution for 1 min dip^−1^. Polysulfide aqueous solution (2.0 m Na_2_S, 2.0 m S, and 0.2 m KCl) was used as the electrolyte.

### Characterization

The surface morphology characterizations and energy dispersive X‐ray spectroscopy (EDS) mapping based on the different materials were analyzed by scanning electron microscopy (SEM, JSM‐IT500A, JEOL) and transmission electron microscopy (TEM, JEM‐F200, JEOL). The crystal structures were determined by X‐ray powder diffractometer (XRD, D8‐ADVANCE, Bruker) and Raman spectra (LabRam HR Evolution, Horiba). X‐ray photoelectron spectroscopy (XPS) studies were recorded with XPS spectrometer (Thermo Fisher ESCALAB XI+). The *J–V* curves were measured using a Keithley 2400 source meter under AM 1.5 G solar simulator with an intensity of 100 mW cm^−2^. Before the test, an NREL standard Si solar cell was used to calibrate the power of the simulated solar light to 100 mW cm^−2^. The photoactive area was 0.159 cm^2^ defined by a black shading mask. External quantum yield (EQE) curves were measured on a Keithley 2000 multimeter under the illumination of a 300 W xenon lamp and a Spectral Product DK240 monochromator. Electrochemical impedance spectroscopy (EIS), Tafel polarization spectroscopy measurements, and cyclic voltammetry (CV) measurements were conducted by an electrochemical system (CHI 760E universal dual potentiostat).

## Conflict of Interest

The authors declare no conflict of interest.

## Author Contributions

T.W. and L.C. contributed equally to this work. S.L. conceived the idea. S.L. and H.S. designed and supervised the work. T.W. and H.S. carried out all of the experiments. L.C. performed the simulation. S.L., H.S., T.W., H.M. L.L., G.Z., and C.X. wrote and revised the manuscript. All authors are involved in data interpretation and commented on the manuscript.

## Supporting information



Supporting Information

## Data Availability

The data that support the findings of this study are available from the corresponding author upon reasonable request.
