# Supplementary material for: In Situ Growth of MoS2 Onto Co‐Based MOF Derivatives Toward High‐Efficiency Quantum Dot‐Sensitized Solar Cells
Source: Adv Sci (Weinh). 2024 Sep 16;11(42):2406476. doi: 10.1002/advs.202406476 (PMC11558139; doi:10.1002/advs.202406476)
Supplement: Supplementary file 1 — Supporting Information [file ADVS-11-2406476-s001.docx]

**Supporting Information**

**In Situ Growth of MoS_2_ onto Co-based MOF Derivatives Toward High-Efficiency Quantum Dot-Sensitized Solar Cells**

Tianming Wang^1,2,#^, Lejuan Cai^2, #^, Caijuan Xia^1,^ *, Han Song^2,3,^*, Lianbi Li^1^, Gongxun Bai^4^, Nianqing Fu^5^, Lede Xian^2^, Rong Yang^6^, Haoran Mu^2^, Guangyu Zhang^2^, Shenghuang Lin^2,^ *

^1^School of Science, Xi’an Polytechnic University, Xi’an, Shanxi 710048, China

^2^Songshan Lake Materials Laboratory, Dongguan, Guangdong 523808, China

^3^College of Chemistry and Chemical Engineering, Xinjiang Normal University, Xinjiang Uygur Autonomous Regions, Urumuqi 830054, China

^4^Key Laboratory of Rare Earth Optoelectronic Materials and Devices of Zhejiang Province, China Jiliang University, Hangzhou 310018, China

^5^ School of Materials Science and Engineering, South China University of Technology, Guangzhou, China.

^6^Changsha Semiconductor Technology and Application Innovation Research Institute, College of Semiconductors (College of Integrated Circuits), Hunan University, Changsha 410082, China

^#^These authors contributed equally to this work.

**Correspondence**

Shenghuang Lin

Songshan Lake Materials Laboratory, Dongguan, Guangdong 523808, China

E-mail: [linshenghuang@sslab.org.cn](mailto:linshenghuang@sslab.org.cn)

**
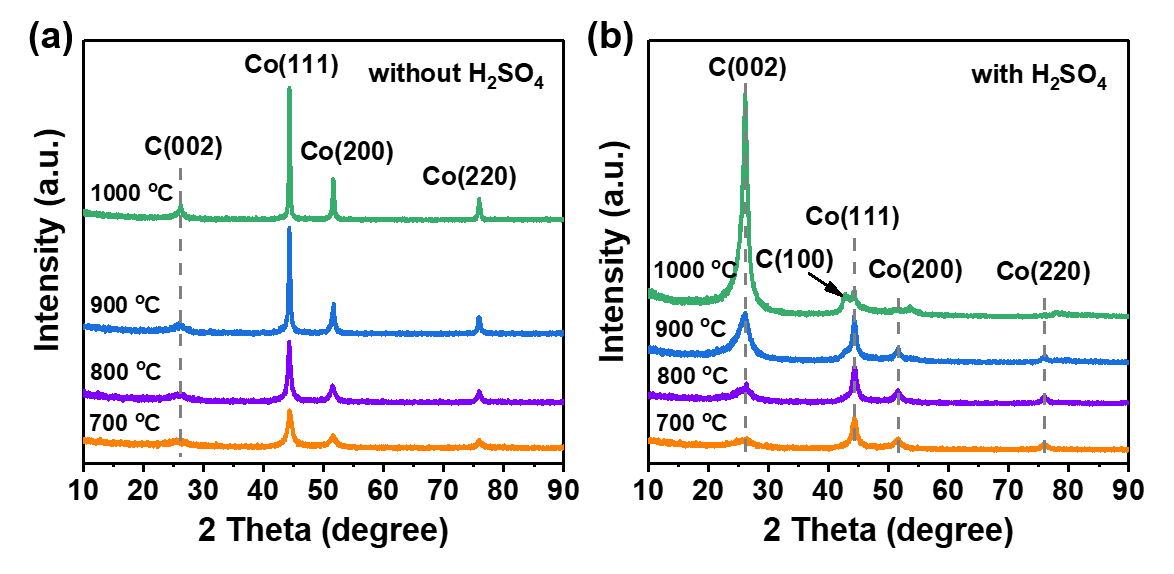
**

**Figure S1.** XRD patterns of Co,N-C without (a) and with (b) H_2_SO_4_ treatment.


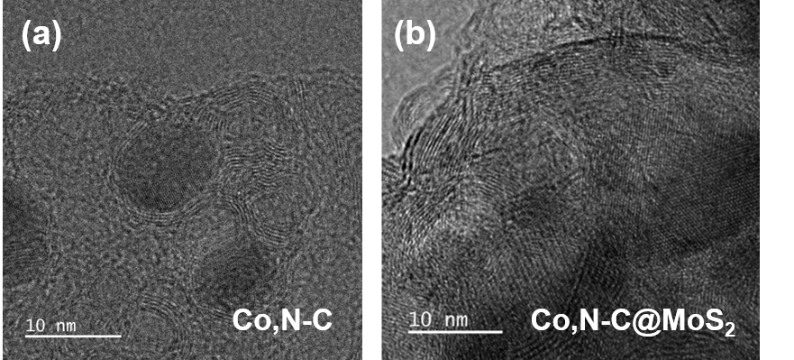


**Figure S2.** TEM images with high resolution of Co,N-C and Co,N-C@MoS_2_.


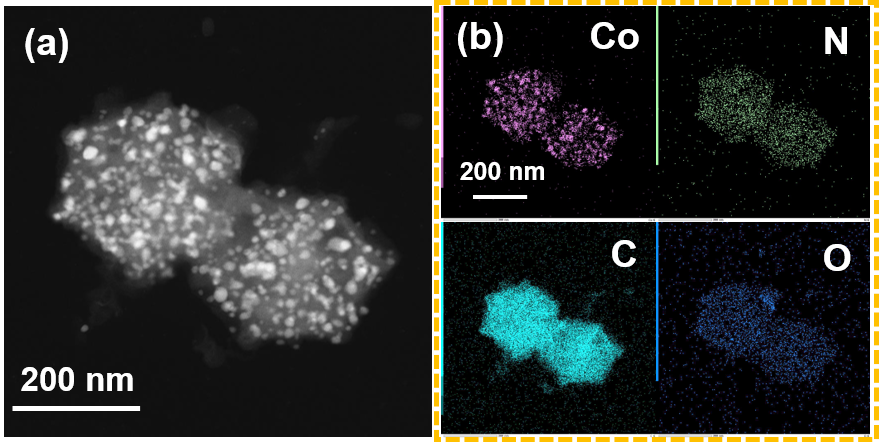


**Figure S3.** (a) TEM and (b) the corresponding EDS-mapping of Co,N-C materials.


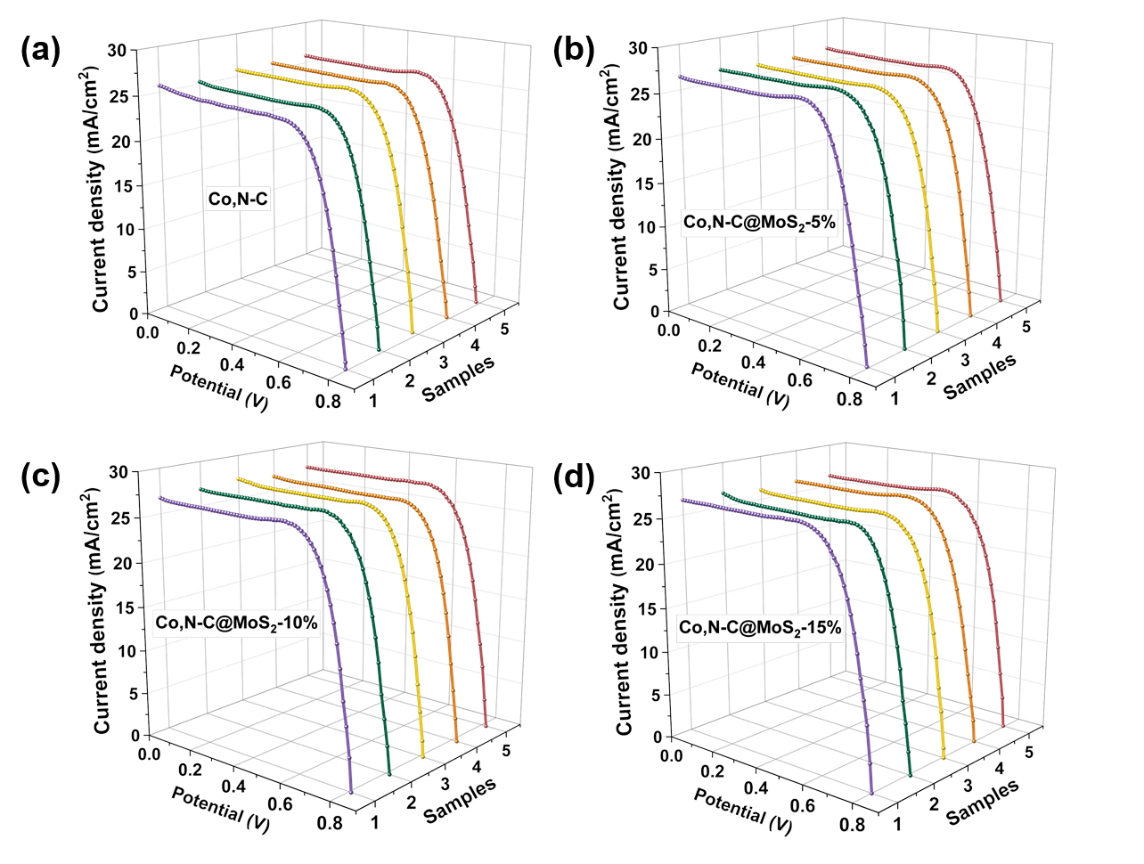


**Figure S4.** *J*-*V* curves (a-d) of ZCISSe QDSCs based on different CEs under the illumination of 1 full sun intensity (AM 1.5G, 100 mW/cm^2^).

**
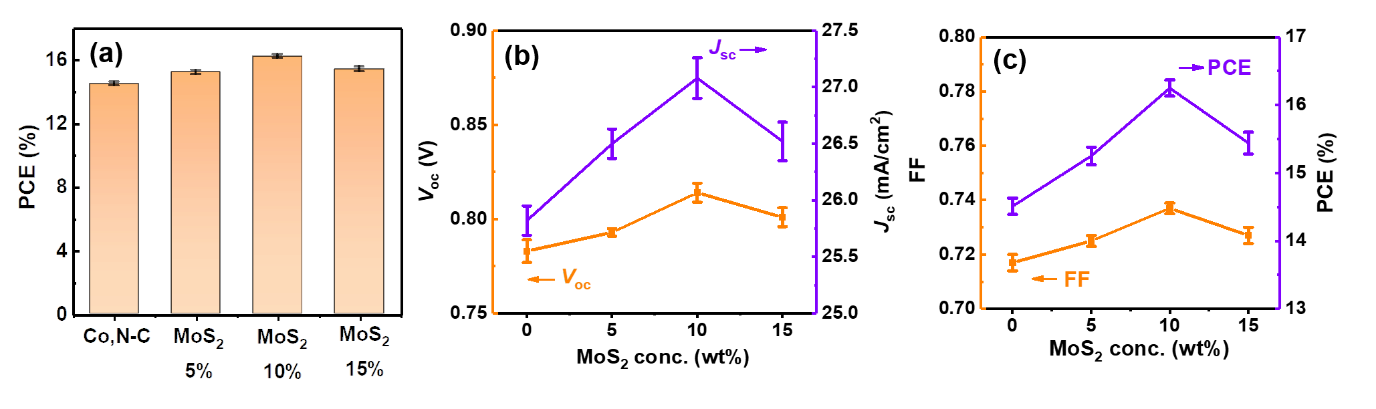
**

**Figure S5.** (a) Summary of PCE values from *J-V* curves of ZCISSe QDSCs based on different Co,N-C@MoS_2_/Ti CEs; Dependence of (b) average *V*_oc_, *J*_sc_; (c) FF and PCE on the concentration of MoS_2_.


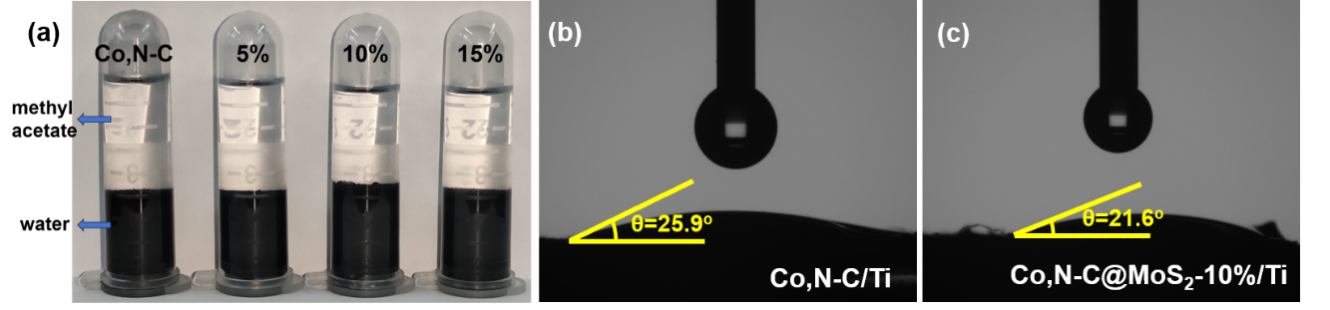


**Figure S6.** (a) The hydrophily ability of Co,N-C@MoS_2_ composite based on different doping amounts of MoS_2_ nanoparticles; contact angles for (b) Co,N-C/Ti and (c) Co,N-C@MoS_2_/Ti CEs.

*
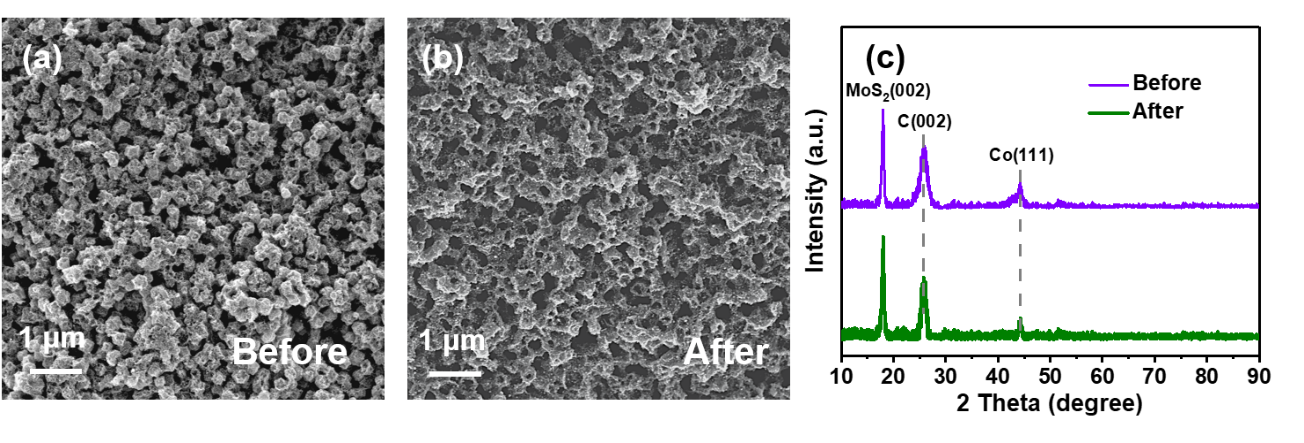
*

**Figure S7.** SEM and XRD pattern of Co,N-C@MoS_2_ composite before and after stability testing.


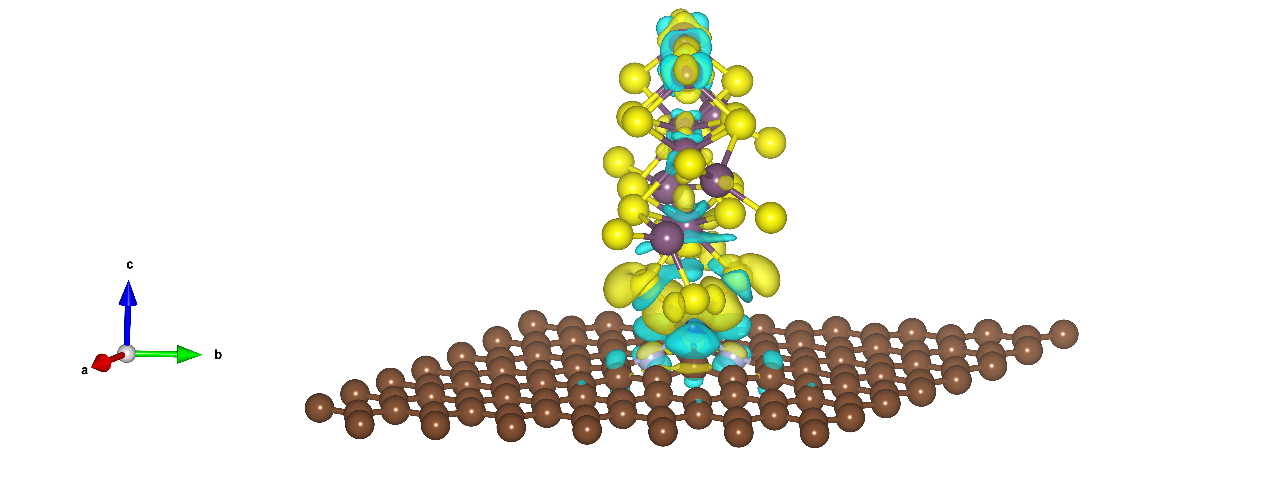


**Figure S8.** The interfacial charge transfer caused by the combination of MoS_2_ and Co,N-C to form Co,N-C@MoS_2_. Yellow and cyan represent electron accumulation and electron depletion, respectively. The isosurface value is 0.002 e/Bohr^3^.

**Figure S9.** The charge density difference plot for Na_2_S_6_ adsorption on the surface of (a) Co,N-C@MoS_2_ and (b) Co,N-C. Yellow and cyan represent electron accumulation and electron depletion, respectively. The isosurface value is 0.0015 e/Bohr^3^ and 0.015 e/Bohr^3^ for Co,N-C@MoS_2_ and Co,N-C, respectively.

**Table S1** Comparison of current density for ZCISSe QDSCs based on different CEs.

| **CEs** | **Integral values**  **(mA/cm^2^)** | **Experimental data**  **(mA/cm^2^)** |
| --- | --- | --- |
| Co,N-C/Ti | 24.73 | 24.84 |
| Co,N-C@MoS_2_-5%/Ti | 25.25 | 25.35 |
| Co,N-C@ MoS_2_-10%/Ti | 25.69 | 25.95 |
| Co,N-C@ MoS_2_-15%/Ti | 25.72 | 25.93 |

**Table S2** Summary of the representative photovoltaic performance for QDSCs based on different CEs.

| **QDs** | **CE Materials** | ***J*_sc_ (mA/cm^2^)** | ***V*_oc_(V)** | **FF** | **PCE**  **(%)** | **Refs** |
| --- | --- | --- | --- | --- | --- | --- |
| CdSeTe | Cu_x_S/C Nss | 20.36 | 0.698 | 0.661 | 9.39 | [1] |
| CdS/CdSe/ZnS | NiCo_2_S_4_ | 16.68 | 0.489 | 38.52 | 3.14 | [2] |
| CdSe | HMII-Co@N-C | 19.00 | 0.686 | 0.53 | 6.85 | [3] |
| CuInGaSe | MC | 25.01 | 0.740 | 0.621 | 11.49 | [4] |
| CdSeTe | CGH/Cu_2_S | 22.65 | 0.769 | 65.63 | 11.51 | [5] |
| CdSeTe | GH-CuS | 20.69 | 0.786 | 66.02 | 10.74 | [6] |
| ZnCuInSe | CNT-CuS | 26.22 | 0.718 | 60.93 | 11.47 | [7] |
|  | GH-CuS | 26.74 | 0.704 | 62.51 | 11.77 |  |
|  | CNT-GH-CuS | 26.87 | 0.782 | 66.70 | 14.02 |  |
| ZnCuInSe | MC | 24.72 | 0.764 | 60.60 | 11.44 | [8] |
|  | N-MC | 25.53 | 0.758 | 63.20 | 12.23 |  |
| ZnCuInSe | MC | 25.97 | 0.752 | 64.40 | 12.57 | [9] |
| ZCISe/CdSe | MC | 27.32 | 0.745 | 62.10 | 12.65 | [10] |
| ZnCuInSe | MC | 25.25 | 0.739 | 62.20 | 11.61 | [11] |
| CdS/CdSe/ZnSe | MoS_2_ | 15.03 | 0.586 | 44.00 | 3.92 | [12] |
|  | MoS_2_-F127 | 17.63 | 0.607 | 56.00 | 6.03 |  |
| CdSe/CdS | NCNT | 14.37 | 0.38 | 41.00 | 2.24 | [13] |
|  | NPCN | 14.09 | 0.43 | 54.00 | 3.27 |  |
| CdSeTe | AC | 19.72 | 0.754 | 66.50 | 9.88 | [14] |
|  | MC | 20.69 | 0.807 | 68.90 | 11.51 |  |
| ZnSe/CdSe | MC | 15.32 | 0.638 | 52.50 | 5.08 | [15] |
| ZnCuInSe | N-MC | 25.50 | 0.756 | 64.70 | 12.47 | [16] |
|  | Co,N-C | 25.75 | 0.787 | 67.80 | 13.74 |  |
| CdS/CdSe | MCNT | 17.75 | 0.455 | 55.70 | 4.50 | [17] |
|  | PbS/MCNT | 17.12 | 0.545 | 68.50 | 6.39 |  |
| ZCISSe | Co,N-C@MoS_2_ | 27.08 | 0.814 | 0.737 | 16.25 | This work |

HMII: 1-hexyl-3-methylimidazolium iodide; MOF: Metal-Organic Frameworks; MC: Mesoporous carbon; CGH/Cu_2_S: chemical graphene hydrogel/Cu_2_S; GH: graphene hydrogel; CNT: carbon nanotube; NCNT: nitrogen-doped carbon nanotubes; NPCN: nitrogen-doped porous carbon nanoribbons; AC: activated carbon; MCNTs: multi-walled carbon nanotubes

**References**

1. M. Chen, F. Yin, Z. Du, Z. Sun, X. Zhou, X. Bao, Z. Pan, J. Tang, *J. Colloid Interface Sci.* **2022**, 628, 22.
2. J. Deng, M. Wang, X. Song, Z. Yang, Z. Yuan, *Nanomaterials* **2018**, 8, 251.
3. M. Li, L. Fu, X. Zhou, C. Shi, H. Hao, J. Jia, *Adv. Energy Sust Res.* **2024**, 5, 2300154.
4. W. Peng, J. Du, Z. Pan, N. Nakazawa, J. Sun, Z. Du, G. Shen, J. Yu, J. Hu, Q. Shen, X. Zhong, *ACS Appl. Mater. Interfaces* **2017**, 9, 5328.
5. X. Ji, C. Yang, W. Fang, H. Zhang, *Electrochim. Acta* **2019**, 297, 980.
6. H. Zhang, C. Yang, Z. Du, D. Pan, X. Zhong, *J. Mater. Chem. A* **2017**, 5, 1614.
7. H. Zhang, X. Ji, N. Liu, Q. Zhao, *Electrochim. Acta* **2019**, 327, 134937.
8. S. Jiao, J. Du, Z. Du, D. Long, W. Jiang, Z. Pan, Y. Li, X. Zhong, *J. Phys. Chem. Lett.* **2017**, 8, 559.
9. L. Zhang, Z. Pan, W. Wang, J. Du, Z. Ren, Q. Shen, X. Zhong, *J. Mater. Chem. A* **2017**, 5, 21442.
10. W. Wang, W. Feng, J. Du, W. Xue, L. Zhang, L. Zhao, Y. Li, X. Zhong, *Adv. Mater.* **2018**, 30, 1705746.
11. J. Du, Z. Du, J. Hu, Z. Pan, Q. Shen, J. Sun, D. Long, H. Dong, L. Sun, X. Zhong, L. Wan, *J. Am. Chem. Soc.* **2016**, 138, 4201.
12. Z. Tian, Q. Zhong, Q. Chen, *Chem. Eng. J.* **2020**, 396, 125374.
13. W. Dong, J. Liu, G. Wang, *Int. J. Energy Res.* **2020**, 44, 6522.
14. Z. Du, Z. Pan, F. Santiago, K. Zhao, D. Long, H. Z, Y. Zhao, X. Zhong, J, Yu, J. Bisquert*,* *J. Phys. Chem. Lett.* **2016**, 7, 3103.
15. Z. Du, M. Liu, Y. Li, Y. Chen, X. Zhong, *J. Mater. Chem. A* **2017**, 5, 5577.
16. Y. Lin, H. Song, H. Rao, Z. Du, Z. Pan, X. Zhong, *J. Phys. Chem. Lett.* **2019**, 10, 4974.
17. Q. Pei, Z. Chen, S. Wang, D. Zhang, P. Ma, S. Li, X. Zhou, Y. Lin, *Solar Energy* **2019**, 178, 108.
